# *In Utero* Hepatocellular Transplantation in Rats

**DOI:** 10.1155/2013/562037

**Published:** 2013-08-24

**Authors:** Emma Muñoz-Sáez, Estefanía de Munck, Paloma Maganto, Cristina Escudero, Begoña G. Miguel, Rosa María Arahuetes

**Affiliations:** ^1^Department of Animal Physiology II, Complutense University of Madrid, C/Jose Antonio Novais 2, 28040 Madrid, Spain; ^2^Experimental Surgery Department of Hospital Universitario Puerta de Hierro Majadahonda, 28222 Madrid, Spain; ^3^Department of Biochemistry and Molecular Biology I, Complutense University of Madrid, C/Jose Antonio Novais 2, 28040 Madrid, Spain

## Abstract

This work represents a step forward in the experimental design of an *in utero* hepatocellular transplantation model in rats. We focused on the enrichment optimization of isolated fetal hepatocytes suspension, arranging the surgery methodology of *in utero* transplantation, monitoring the biodistribution of the transplanted hepatocytes, and assessing the success of the transplants. Rat fetuses have been transplanted at the 17th embryonic day (ED17) with fetal hepatocytes isolated from rats at the end of pregnancy (ED21). We assessed possible differences between lymphocyte population, CD4 positive, CD8 positive, double-positive T-cells, and anti-inflammatory cytokines interleukins 4 and 10 (IL4 and IL10) as well. Cellular viability reached the rates of 90–95%. Transplanted groups had a limited success. Transplanted hepatocytes were not able to pass through the hematoplacental barrier. The hepatocytes injected were primarily located in the liver. There was an upward trend in the whole amount of T CD4 and T CD8 cells. There was an increased IL4 in the transplanted groups observed in the pregnant rats. The possibility to induce tolerance in fetuses with a hepatocyte transplant *in utero* could be a key point to avoid the immunosuppression treatments which must be undergone by transplanted patients.

## 1. Introduction 

Terminal hepatic diseases are the final consequence of many chronic liver diseases, damaging irreversibly its functions [1, 2]. The prevalence of these pathologies in Europe is estimated to represent about 6% of the whole number of diseases. 

Classically, these hepatic diseases have been treated by means of orthotopic liver transplantation [3]. Nevertheless, the balance between advantages and risks of this therapy must be evaluated, and unfortunately the number of patients who can benefit is very limited [3], and this procedure is associated with several problems [4].

The idea of using hepatocytes or fragments of liver as a treatment was proposed for the first time by Eiseman in 1967 [5]. Thus, the cellular transplantation using hepatocytes is an emergent field in the clinical therapy to treat hepatic disorders [6]. Since the first hepatocellular transplant in 1992 [3], more than 80 patients have been treated using this approach [7]. Moreover, the transplantation of hepatocyte mass equivalent to 10% of the patients' livers should be sufficient to normalize the metabolic situation [8]. The cellular transplant has some advantages: it is cheaper, it is a less invasive technique, it is associated with smaller mortality and morbidity, and it is less immunogenic [8]. 

It has been demonstrated that adult hepatocytes have a limited capacity to proliferate [9]. In contrast, the early fetus is exceptionally tolerant to foreign antigens accepting cells even when the major histocompatibility complex (MHC) does not match [6, 10]. In addition, the transplantation of cells *in utero* to cure fetuses with birth defects has several advantages [11] and does not involve the immune system of the mother [12]. In fact, *in utero* transplantation has been performed in different animal models besides the rat, like for instance sheep [13], chimpanzees [14], and pigs [15]. 

Since it is difficult to culture mature hepatocytes *in vitro*, cells derived from other tissues are also being investigated as potential candidates in some hepatic diseases [16]. There are different animal models that express as a marker an enzymatic deficiency to differentiate the isolated and transplanted cells from the host's hepatocytes [6] like the Gunn rat model [17] and the most used model F344 rats [18]. 

The possibility of generating the induction of tolerance with an *in utero* transplantation of hepatocytes is a key point to avoid the immunosuppression treatments undergone by the transplanted people [19]. The majority of studies have focused on T cells as the most important effectors of this response, including T CD4 and CD8 [20]. The condition to achieve a successful transplantation consists of inducing immunological tolerance to the grafts [21, 22]. Therefore, the hepatocellular transplantation can be an option for patients with inherited metabolic diseases [8, 20].

For these reasons, we develop a model of hepatocellular transplantation *in utero* to avoid immunosuppression in future treatments. Our aims are to optimize the enrichment of the suspension of isolated fetal hepatocytes, arranging the surgery methodology of *in utero* transplantation, monitoring the biodistribution of the transplanted hepatocytes, assessing the success of the transplants, to analyze the lymphocyte populations (T CD4 and T CD8 cells), and to analyze the anti-inflammatory cytokines (interleukins 4 and 10).

## 2. Materials and Methods 

### 2.1. Experimental Animals

Pregnant females of the *Rattus norvegicus* species, albinus variety, Wistar, and Fischer (F344) strains (Harlan Laboratories) were used. Animal donors were fetuses on day 21 of gestation (ED21), and the hepatocellular transplantation is performed in fetuses on day 17 (ED17). In order to determine the day of gestation, the rats were mated in a room with controlled photoperiod cycles (12 hours light and 12 hours darkness from 8:00 a.m. to 15:00 p.m.). Next, a vaginal smear was taken and visualized under optical microscope looking for the presence of sperm. If smears were positive, that was considered the day 0 of gestation (ED0) from which the days of gestation for the animal donors and the receivers were counts. The rats were maintained in the Animal Facility of the Faculty of Biological Sciences of the Complutense University of Madrid under controlled photoperiod conditions, with 12 hours light and 12 hours darkness per day. In any case, food and water were supplied ad libitum. Animals were maintained in accordance with the principles set forth in the National Institute of Health (NIH) guide for the care and use of laboratory animals.

### 2.2. Experimental Groups

**Figure 1:**
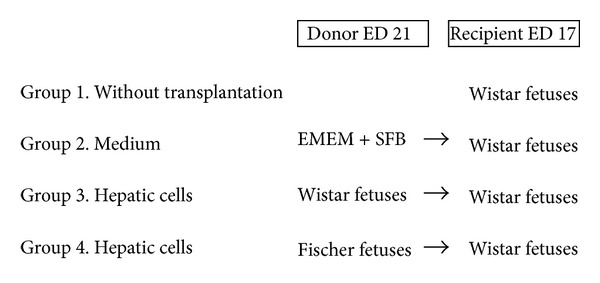
Scheme of the four experimental groups (group 1:  *n* = 60, group 2:  *n* = 113, group 3:  *n* = 153, and group 4:  *n* = 163).

Four experimental groups were made as described in Figure 1. No surgery was carried out to those pregnant rats belonging to group 1. The second experimental group was transplanted with 10 *μ*L EMEM + SFB without cells. For group 3, the hepatocytes of Wistar fetuses were isolated at embryonic day 21 and subsequently resuspended in 10 *μ*L medium; for transplantation to Wistar fetuses on ED17: 10^6^, cells were transplanted to them. In contrast, in the fetuses included in group 4, the hepatocytes were isolated from Fischer fetuses on day 21 of gestation and afterwards transplanted *in utero* to fetuses of Wistar rat on embryonic day 17.

### 2.3. Fetal Hepatocytes Isolation

After anesthetizing the pregnant rat at ED21 with 4% isoflurane in O_2_, a medial laparotomy by planes was done, both uterine horns were externalized, and the fetuses were extracted removing the vitelline membrane and the placenta. Next, the fetuses were sacrificed, and the livers were obtained with curved tweezers by mechanical traction in the abdominal region of the fetus. The isolation of fetal hepatocytes was carried out according to the protocol described by Berry and Friend [23] and modified by Arahuetes et al. [24]. The suspension of cells obtained was filtrated through a mesh of Nylon of 50 *μ*m pore size, then it was centrifuged at 1000 g for 5 minutes at 4°C, and the supernatant was discarded. An ammonium chloride buffer, or lysis buffer (NH_4_Cl 0,155 M, KHCO_3_ 0,01 M, EDTA 10–6 M, add distilled water to 500 mL), was added to the pellet obtained. With this method, the hepatocytes suspension is enriched since the erythrocytes are lysates. Lysis was carried out in ice, adding 0.5 mL lysis buffer per 1 mL cellular suspension, followed by an incubation during 5 minutes. The reaction was stopped adding the same volume used for the lysis buffer of EMEM + bovine fetal serum (BFS). Afterwards, the suspension was centrifuged at 1000 g for 1 minute at 4°C, the supernatant was discarded, and the pellet was washed twice with EMEM + BFS and eventually resuspended in a known volume.

The percentage of hepatocytes in this suspension was around 70% in all cases. As the purification with biomagnetic technics damaged the hepatocyte membrane, we assumed that the major part of our suspension were fetal hepatocytes [25].

Then, the cellular viability was tested by exclusion of the vital dye trypan blue (0.2%), and the cells were counted in a Neubauer chamber. It is important that the suspension of fetal hepatocytes remains in ice until the moment of transplantation. 

### 2.4. Transplanted Fetal Hepatocyte 111In-Oxine Labeled and Biodistribution

In order to track the transplanted hepatocytes, they were labeled incubating them 30 minutes at 37°C with 14.8 MBq 111Indium-oxine. The 111Indium linked the intracellular proteins as a result of oxina which promotes the entrance of 111Indium-oxine at intracellular section. This was not a specific marker, all of our cells were going to express the 111In-oxine. Since we were interested in cell homing over several days, we selected 111In as the radionuclide with a half-life of 67 h [26].

The cells were transplanted as described in the following item. Gammagraphic images of the pregnant rats and the fetuses were obtained at 6, 24, and 48 hours posttransplantation. Later, they were sacrificed, and placentas as well as liver, spleen, heart, lungs, and digestive apparatus of the fetuses were collected.

### 2.5. Methodology of the Hepatocellular Transplantation

**Figure 2:**
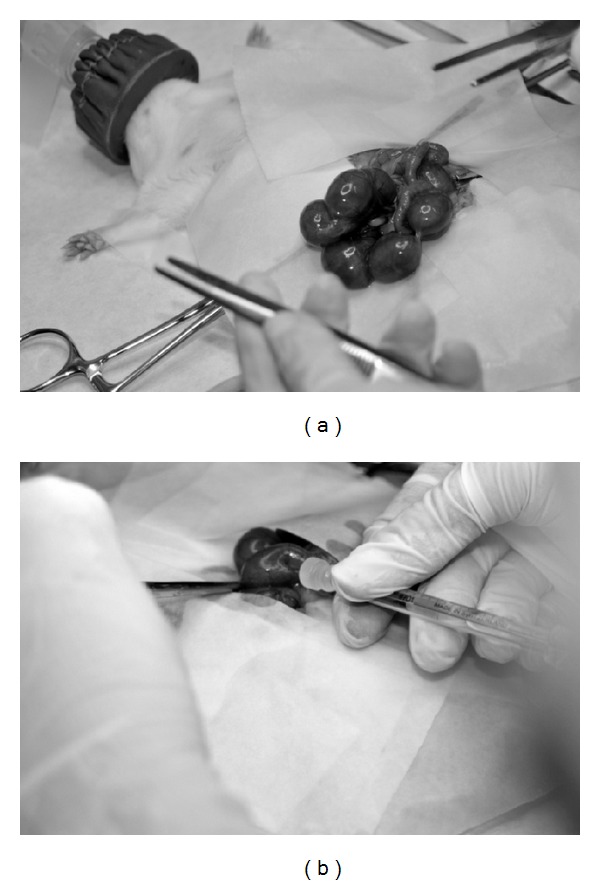
Pictures taken during transplantation. (a) A uterine horn can be observed; in this step, the disposition of the fetuses in the uterus was visualized. (b) The place where the intraperitoneal injection to a fetus *in utero* was made is shown.

The pregnant rat was anaesthetized at ED17 with 4% isofluorane in O_2_ and placed on a heating blanket at a sustained temperature of 37-38°C. Next, a medial laparatomy by planes was performed trying to prevent a long incision, in order to externalize uterine horns. However, the operation was carried out horn by horn, maintaining it always moisturized and at 37°C. The disposition of the fetuses was determined, and 10^6^ fetal hepatocytes (contained in 10 *μ*L EMEM SFB) were injected to them through uterus with a Hamilton hypodermic syringe of 10 *μ*L of 30 G (6 pk 30/10 mm7pst2) and intraperitoneally in the fetuses, that is, between the hindlimb and the dark area which represents the fetal liver (Figure 2). Once all fetuses were transplanted, the uterine horn is reintroduced into the abdominal cavity, and we proceed the same way with the other one. Finally, transplanted fetuses were counted (to contrast the viability of the surgery with respect to the newborn), and the pregnant rat was sutured with continuous stitches in the muscular layer and discontinuous stitches in the skin layer. Both sutures were completed with absorbable braided thread.

### 2.6. Sample Collection

At postnatal day 15 (P15), blood and liver samples were taken and the pups were sacrificed. The liver samples were frozen to −80°C, and the blood was separated into two aliquots. One of the aliquots was kept at room temperature, as complete blood sample, to assess the lymphocyte populations by flow cytometry. The other one was centrifuged to obtain serum, which was frozen to −20°C before quantifying interleukins 4 and 10.

### 2.7. Assessment of Lymphocyte Populations

The complete blood aliquot was divided into three subaliquots of 100 *μ*L each, to carry out flow cytometry analysis. First, each subaliquot underwent lysis, at room temperature and in the dark, using BD FACS Lysis solution (BD Bioscience). Then we labeled the different populations of lymphocytes with antibodies, following manufacturer indications. The first aliquot is used as negative control, without any additional antibody. The second one contains mouse antiRat-CD4 FITC/CD8 RPE (AbD Serotec) and mouse antiRat-CD3APC (BD Bioscience, Pharmigen). The third one contains mouse antiRat-CD4 FITC/CD25 RPE (AbD Serotec) and mouse antiRat-CD3 APC (BD Bioscience, Pharmigen). Then the labeled populations were determined by flow cytometry.

### 2.8. Interleukin Assessment

The interleukins 4 and 10 were quantified in the serum samples collected from the pups at P15. A commercial ELISA in 96-well plate was done, Rat IL4 or IL10 Platinum ELISA (eBioscience), following the manufacturer's protocol. A standard curve was constructed for each cytokine using the suitable standards. The absorbance at 450 nm is displayed in the *y*-axis and concentrations (in pg/mL) in the *x*-axis. 

The following standard curves were obtained. For IL4; *y* = 3,792*e* ^−0,56*x*^; *R* ^2^ = 0,993. For IL10:*y* = 2,417*e* ^−0,79*x*^; *R* ^2^ = 0,969.

When the results of absorbance (*y*) yielded by each sample is extrapolated in these equations, the concentration of IL4 or 10 in pg/mL (*x*) of the sample can be obtained.

### 2.9. Statistical Analysis

The results were processed with the GraphPad Prism 4 software, selecting variables within a single group, and performing one-way ANOVA with normalized data. For repeated and nonparametric measurements, the Friedman's test was used. The data were considered significant if the yielded *P* value was smaller than 0.05.

## 3. Results 

### 3.1. Hepatocyte Enrichment and Viability

**Figure 3:**
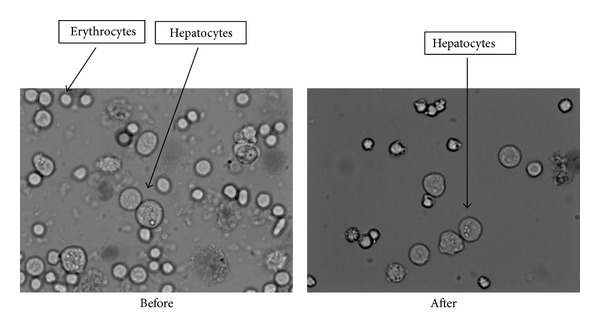
Pictures of the cellular suspension dyed with trypan blue and visualized with an optical microscope. We can see how the suspension looks before and after the lysis. Hepatocytes are bigger and have vesicles in their cytoplasm, while erythrocytes are smaller, round, and refringent.

The number of cells obtained in every isolation was around 15 × 10^6^ cells/liver weight (g), and the average viability was about 90–95% (Figure 3).

### 3.2. Hepatocellular Transplantation Viability

In the case of individuals without transplantation, the total newborn pups coincide with the born-alive pups. This is logical, because they have not suffered surgery of any kind. The transplantations performed with medium show the negative effect the surgery can have, since there is a slight decrease in the number of newborn pups and born-alive pups with respect to the number of transplanted fetuses. So that the possible damage the vehicle in which cells are re-suspended could cause is discarded. In the transplantation groups 3 and 4, the total offspring and the born-alive pups decreased considerably to yield significant differences (*P* = 0.02 for both groups).

### 3.3. Tracking of Labeled Hepatocytes

**Figure 4:**
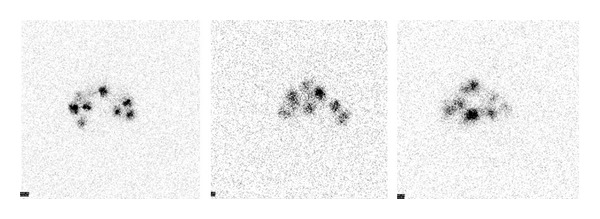
Gammagraphic images of the pregnant rats. There are deposits of 111In-oxine in the abdominal cavity in similar number to transplanted fetuses.

The gammagraphic images of the pregnant rats exhibit important deposits of 111In-oxine in the abdominal cavity, similar in number to the number of transplanted fetuses (Figure 4). 

**Figure 5:**
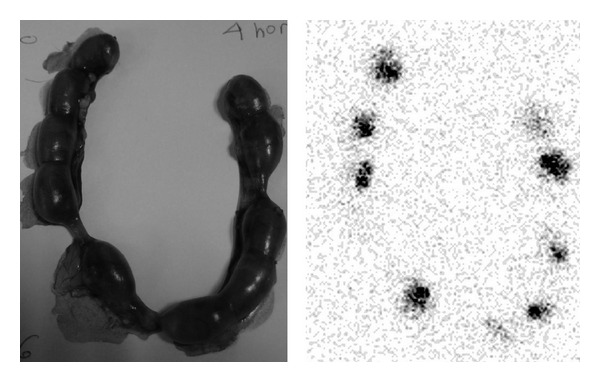
Gammagraphic image of the uterus *ex vivo*. Different fetal deposits of 111In-oxine are clearly visualized after transplantation with labeled hepatocytes.

Gammagraphic images of the uterus *ex vivo* revealed more clearly the different fetal deposits (Figure 5). Gammagraphic images of the rats after uterus extraction did not show any deposit (image not shown). There was no significant activity in other organs from the pregnant rat. Nevertheless, the evaluation of the placentas showed significant activity. Also, the intensity of the deposits decreased while the implanted cells proliferate.

**Figure 6:**
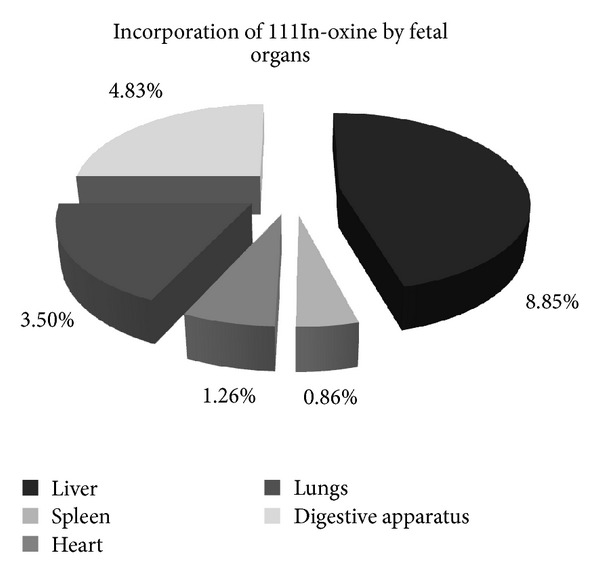
Incorporation of 111In-oxine by different fetal organs. Hepatocytes were mostly located in the liver. There were other deposits found at the digestive apparatus, lungs, heart, or spleen, although the proportion of these was smaller than the amount in the liver.

The gammagraphic images taken from different fetal organs had little or no activity, except for some isolated livers and spleens. The activity was determined for each organ (percentage of 111In-oxine incorporated by the organ with respect to the total amount in the fetus). Livers incorporate more amount than spleen, heart, lung, and digestive apparatus (Figure 6). The rest of the body and placentas exhibited a high activity. 

### 3.4. Assessment of Lymphocyte Populations

**Figure 7:**
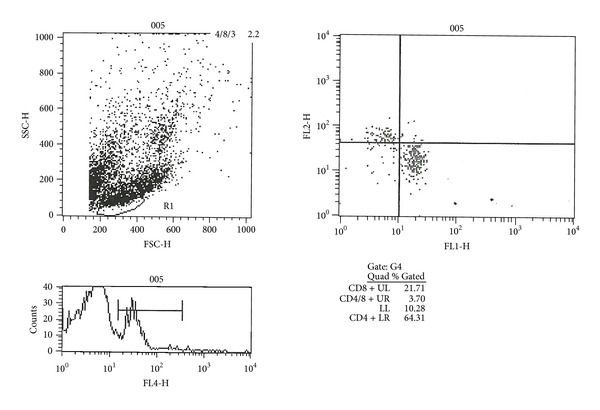
Example of one of the flow cytometry analyses where the percentages of each analyzed lymphocyte population (of a pup at postnatal day 15 belonging to the experimental transplantation group 3) are shown.

Here, we show the individual results of one of the flow cytometry analyses (Figure 7).

The averages of all the analyzed samples were calculated and graphically represented, detailing the lymphocyte population studied and the percentage of positive cells for that population in each experimental group (including in the group both mothers and pups). There were no significant differences between groups and the lymphocyte populations analyzed in the case of mothers (data not shown). In the pups there were no significant differences either, although a trend can be seen.

**Figure 8:**
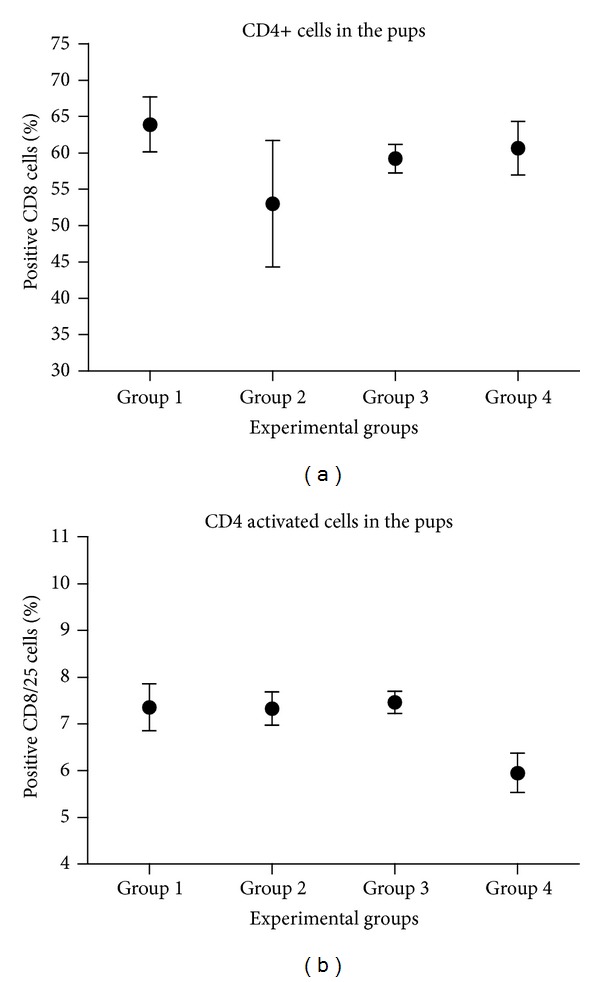
Evaluation of the total CD4 positive lymphocyte population (a) and the activated population (b). In the *x*-axis, it showed the experimental groups and in the ordinates the average percentage of positive CD4 cells without activating or activated with his bar of error.

The population of positive lymphocytes T CD4 does not show significant differences in any of groups of treatment in the newborn ones. The same happens in the case of activated lymphocytes T CD4 (Figure 8).

**Figure 9:**
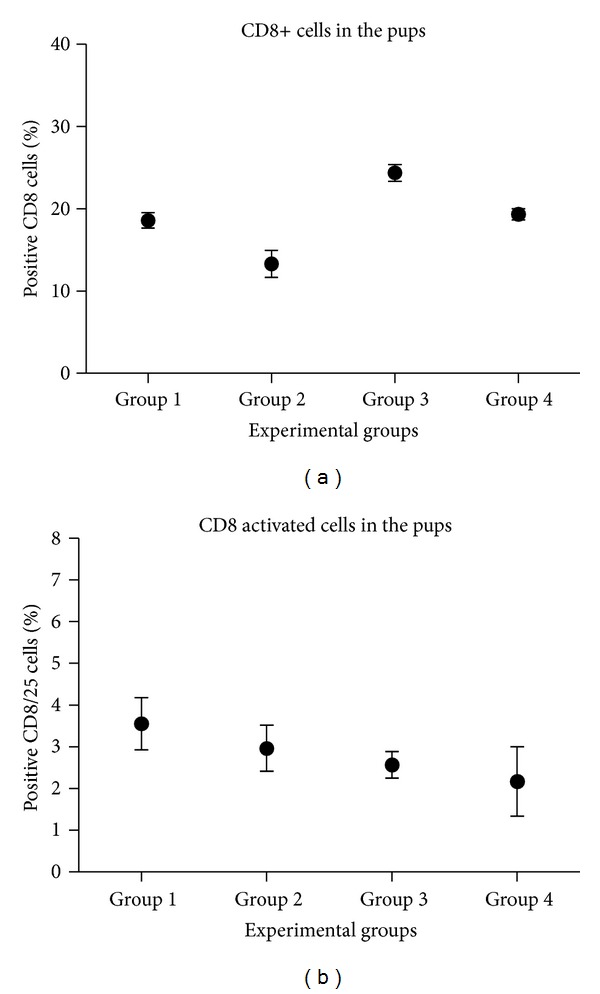
Assessment of total CD8 positive lymphocyte population (a) and the activated population (b). The experimental groups are displayed in the *x*-axis, and in the *y*-axis, the average percentage of positive CD8 cells is displayed.

No statistically significant differences were observed between groups of pups for total and activated T CD8 lymphocytes. In this case, there is no relevant significance between experimental groups either (Figure 9).

**Figure 10:**
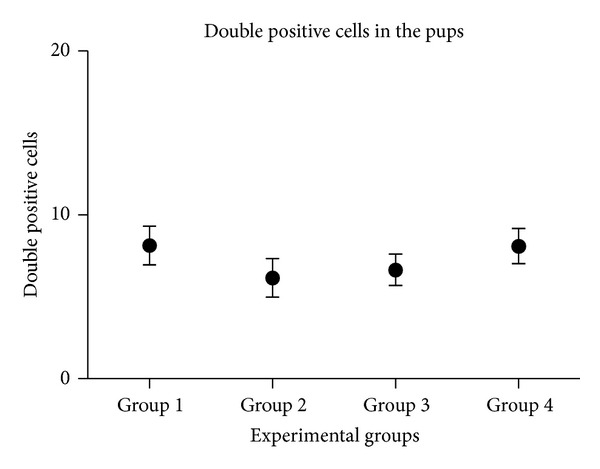
Double positive lymphocyte population. The experimental groups are displayed in the *x*-axis it showed, and in the *y*-axis, the average percentage of double positive cells is displayed.

The percentage of double positive lymphocyte cells (i.e., positive for both CD4 and CD8) is very low, although there are no significant differences between the different experimental groups (Figure 10). 

### 3.5. Interleukin Assessment in Serum

**Figure 11:**
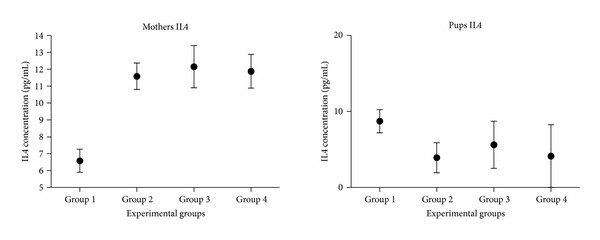
Quantification of interleukin 4 in the serums of mothers and pups. In the *x*-axis, the different experimental groups are displayed; in the *y*-axis, the average concentration of IL4 in pg/mL is represented.

**Figure 12:**
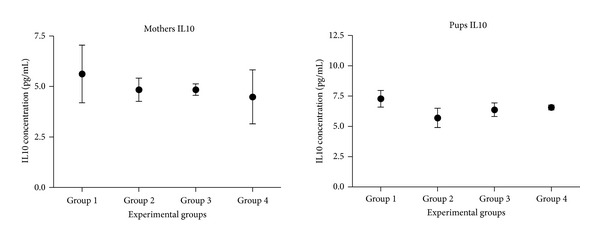
Quantification of interleukin 10 in the serums of mothers and pups. In the *x*-axis, the different experimental groups are displayed; in the *y*-axis, the average concentration of IL10 in pg/mL is represented.

IL4 and the IL10 were quantified both in mothers and transplanted pups. Although there is a clear tendency in the concentration of both interleukins, there were no significant differences between experimental groups (Figures 11 and 12).

The concentration of IL4 in mothers that underwent surgery tends to increase with respect to mothers that were not operated. However, this tendency is not observed in pups (Figure 11).

No significant differences were observed for the concentration of IL10, neither in mothers nor pups between groups (Figure 12).

## 4. Discussion

The hepatocellular viability obtained after the isolation process (approx. 90–95%) is high enough to determine that the cellular suspension is suitable for the transplantation (Figure 3) [27]. However, the success decreases when the injections contain hepatocytes. This could be due to an immune reaction triggered in the recipient fetuses [28]. It has been seen that allogeneic transplantation of hepatocytes in mouse is limited by immunological responses to the cells transplanted, even more when the transplantation is carried out *in utero* [29]. Nevertheless, many studies have reported that group 3 similar transplantations have been better tolerated if the procedure was carried out *in utero* [28].

In this work, we chose fetal hepatocytes instead of adult ones because, in previous studies of our laboratory, we found that these hepatoblasts keep all their potentialities and characteristics of low immunogenicity and high proliferativity [6, 17]. Moreover, the transplantation of cells *in utero* to cure fetuses with birth defects has several advantages; namely, (i) the fast growth of fetuses provides an unique opportunity for the settlement and expansion of the implanted cells; (ii) the fetal immunological immaturity and the potential to induce specific tolerance to the donor; (iii) the protective and sterile fetal environment contributes to isolate environmental pathogens; and (iv) the fact that precocious treatments are beneficial and critical to assure effectiveness [11]. 

The purification of hepatocellular suspensions from fetal rat is justified mainly by two factor; first, the fetal liver has a very important role in hemapoiesis; and second, the contamination of the suspension with blood cells is usual [20]. Previous to the present work, we performed a compared study on the purification of fetal hepatocytes using a biomagnetic isolation and measured the results by flow cytometry [6, 25]. As the cells suffer a lot with the purification, we assume that about 70% of our cell suspensions were hepatocytes.

Determining the location of transplanted hepatocytes is important to assess if the failures in the transplants are due to the erratic situation of the hepatocytes and also to check if transplantation had been correctly performed; that is, if the cells had been injected in the peritoneal cavity of the fetuses (Figure 5). We demonstrated that transplanted hepatocytes to the fetuses are not able to cross the placental barrier, and therefore they do not appear in the pregnant rat (Figures 4 and 5). This fact provides more safety about this type of therapeutic procedures and constitutes a step forward for their clinical application [6]. 

Since most of the transplanted hepatocytes were in the liver, the surgical process was demonstrated to be correct (Figure 6). Suckow et al. [28] showed that, after a hepatocellular transplant *in utero*, the hepatocytes were essentially located in the liver and some of them in the lungs [28], coinciding with our results. The localization of the transplanted hepatic cells is relevant, since the ectopic transplantation of hepatocytes has been reported to have therapeutic effectiveness [6]. We think it would be interesting to evaluate the signals involved in the spatial settlement of the transplanted hepatocytes somehow. Indeed, there were more cellular types apart from fetal hepatocytes in the transplantation mixture. This mixture also can include stem-like cells; this could be another explanation for the biodistribution of the labeled cells.

We also assume that the transplant was successful and our transplanted cells were dividing because the radiolabel signal from deposits of 111In-oxine decreases with increasing time after transplant.

When we assessed the lymphocyte populations in the pups at P15 day, we did not find significant differences in the number of T CD4 and T CD8 positive cells, both total and activated. 

There was an important increase of CD4 positive cells in group 2 (Figure 8). This fact was possible to be explained because the culture medium includes SFB containing a lot of proteins which were able to activate the immune cell response.

A trend to increase can be seen in T CD4 positive cells and total T CD8, in the transplanted groups 3 and 4 (Figures 8(a) and 9(a)). We could not see any significant difference between groups in the double positive cells percentage either (Figure 10). 

Significant differences were not obtained in the quantification of interleukins 4 and 10 between groups. For the IL4, we have seen that there are increased levels of this cytokine in the blood of the mothers of every group (Figure 11). IL4 is an anti-inflammatory cytokine; therefore, its increase after transplantation is beneficial for the animal, because they can avoid the subsequent inflammation. This case only happens in the mothers, because their immune system is completely developed and their Th2 cells, IL4 producers (namely, activated basophils, mast cells, and eosinophils), are functionally active [30]. In the pups, there are smaller levels of IL4. This could lead to more inflammation and therefore could maybe cause (or be one of the causes of) the high morbidity of the transplanted fetuses, because they will not be able to inhibit inflammatory substances such as IL1, TNF*α*, IL6, and the inflammatory protein of the macrophage [31]. It would be interesting to quantify these inflammatory cytokines to contrast this hypothesis.

About the data obtained for the IL10, also known as cytokine synthesis inhibitory factor (CSIF) (Figure 12), no significant differences were found between experimental groups, neither in mothers nor in pups. Since there are no high levels of IL10, it is probable that the balance between Th1 cells and Th2 cells is not altered [30]. Depending on the conditions of the transplant, tolerance or allosensitization can be developed. The factors that may influence the response are: time taken for transplantation, transplanting cell dose and composition, the trauma caused by the prenatal surgery, the condition of the immune system during the cell injection, and the level of postnatally induced chimerism [31]. Moreover, it has been seen that hepatocyte transfusion increases lifespan of rats that undergo a hepatic transplantation and improves the production of hepatic proteins [32]. 

Finally, it is important to mention that this work contributes with interesting tools for hepatocellular transplantation *in utero*. Indeed, we consider that it is necessary to further research in this field in order to achieve a deeper knowledge, because of the relevance to unveil the mechanisms involved in tolerance induction and allosensitization, to allow the development of safe prenatal transplantation protocols making a success in individuals with congenital disorders.

## 5. Conclusion

We obtained rates of 90–95% cellular viability making the hepatocyte suspension isolated from rat fetuses at ED 21 suitable for transplantation. Also, both types of transplantation have a limited success. Indeed, transplanted hepatocytes are not able to pass through the blood-placenta barrier. Moreover, the hepatocytes injected to fetuses of pregnant rats at ED17 were primarily located in the liver, although they show some other ectopic locations. In transplanted experimental groups, there is an upward trend in the whole amount of T CD4 and T CD8 cells in transplanted experimental groups. In addition, there is an increase of IL4 in the transplanted groups in the pregnant rats. As it is an anti-inflammatory cytokine, this increment is positive because it protects the mother from inflammatory processes caused by the surgery.
